# Lexicon Development for COVID-19-related Concepts Using Open-source Word Embedding Sources: An Intrinsic and Extrinsic Evaluation

**DOI:** 10.2196/21679

**Published:** 2021-02-22

**Authors:** Soham Parikh, Anahita Davoudi, Shun Yu, Carolina Giraldo, Emily Schriver, Danielle Mowery

**Affiliations:** 1 School of Engineering and Applied Science University of Pennsylvania Philadelphia, PA United States; 2 Department of Biostatistics, Epidemiology, & Informatics University of Pennsylvania Philadelphia, PA United States; 3 Division of Hematology/Oncology Department of Medicine Hospital of the University of Pennsylvania Philadelphia, PA United States; 4 Philadelphia College of Osteopathic Medicine Philadelphia, PA United States; 5 Data Analytics Center Penn Medicine Philadelphia, PA United States; 6 Department of Biostatistics, Epidemiology, & Informatics Institute for Biomedical Informatics University of Pennsylvania Philadelphia, PA United States

**Keywords:** natural language processing, word embedding, COVID-19, intrinsic, open-source, computation, model, prediction, semantic, syntactic, pattern

## Abstract

**Background:**

Scientists are developing new computational methods and prediction models to better clinically understand COVID-19 prevalence, treatment efficacy, and patient outcomes. These efforts could be improved by leveraging documented COVID-19–related symptoms, findings, and disorders from clinical text sources in an electronic health record. Word embeddings can identify terms related to these clinical concepts from both the biomedical and nonbiomedical domains, and are being shared with the open-source community at large. However, it’s unclear how useful openly available word embeddings are for developing lexicons for COVID-19–related concepts.

**Objective:**

Given an initial lexicon of COVID-19–related terms, this study aims to characterize the returned terms by similarity across various open-source word embeddings and determine common semantic and syntactic patterns between the COVID-19 queried terms and returned terms specific to the word embedding source.

**Methods:**

We compared seven openly available word embedding sources. Using a series of COVID-19–related terms for associated symptoms, findings, and disorders, we conducted an interannotator agreement study to determine how accurately the most similar returned terms could be classified according to semantic types by three annotators. We conducted a qualitative study of COVID-19 queried terms and their returned terms to detect informative patterns for constructing lexicons. We demonstrated the utility of applying such learned synonyms to discharge summaries by reporting the proportion of patients identified by concept among three patient cohorts: pneumonia (n=6410), acute respiratory distress syndrome (n=8647), and COVID-19 (n=2397).

**Results:**

We observed high pairwise interannotator agreement (Cohen kappa) for symptoms (0.86-0.99), findings (0.93-0.99), and disorders (0.93-0.99). Word embedding sources generated based on characters tend to return more synonyms (mean count of 7.2 synonyms) compared to token-based embedding sources (mean counts range from 2.0 to 3.4). Word embedding sources queried using a qualifier term (eg, dry cough or muscle pain) more often returned qualifiers of the similar semantic type (eg, “dry” returns consistency qualifiers like “wet” and “runny”) compared to a single term (eg, cough or pain) queries. A higher proportion of patients had documented fever (0.61-0.84), cough (0.41-0.55), shortness of breath (0.40-0.59), and hypoxia (0.51-0.56) retrieved than other clinical features. Terms for dry cough returned a higher proportion of patients with COVID-19 (0.07) than the pneumonia (0.05) and acute respiratory distress syndrome (0.03) populations.

**Conclusions:**

Word embeddings are valuable technology for learning related terms, including synonyms. When leveraging openly available word embedding sources, choices made for the construction of the word embeddings can significantly influence the words learned.

## Introduction

### Background

COVID-19 has become a pandemic that is felt throughout the world. Scientists are developing new methods for determining infection rates, disease burden, treatment efficacy, and patient outcomes [1]. Our ability to detect and phenotype patients with COVID-19 and controls for clinical and translational studies requires clinical symptomatology, radiological imaging, laboratory tests, and associated disorders derived from electronic health record data [2]. Much of this information can be locked within the electronic health record clinical notes [3]. To accurately characterize each patient’s COVID-19 profile for study, we must develop natural language processing systems to reliably extract COVID-19–related information. One of the first steps to extracting this information is developing lexicons with adequate coverage for all synonyms describing each COVID-19 concept. In the clinical domain, lexicons have been developed using several techniques: standardized vocabularies [4], lexico-syntactic patterns [5], term expansion [6], and distributional semantics [7]. Moreover, word embedding technologies have become increasingly popular for identifying semantically and syntactically-related terms within vector spaces by assessing the *distributional hypothesis* that “words that share a common, relative vector space will often also share a common, semantic relatedness” [7].

### Word Embeddings

Word embeddings represent a word in a vector space while preserving its contextualized usage. Word embeddings have been leveraged to learn synonyms to develop lexicons [8]. These vectors are commonly learned by training algorithms like Word2Vec [9], FastText [10], and global vectors for word representation (GloVe) [11] on large corpora, including domain-independent texts (eg, internet web pages like Wikipedia and CommonCrawl, and social media like Twitter and Reddit) and domain-dependent texts (eg, clinical notes like the Medical Information Mart for Intensive Care III [MIMIC III] database notes [12] and biomedical research articles like PubMed). These domain-dependent embeddings may capture richer biomedical information than domain-independent embeddings (eg, *standard GloVe embeddings*) and are becoming increasingly available to the community at large. For example, *BioASQ* released their embeddings trained using the Word2Vec algorithm on 11 million biomedical abstracts from PubMed [13]. Pyysalo et al [14] trained embeddings using Word2Vec on a combination of PubMed and PubMed Central articles along with Wikipedia to combine open domain and biomedical knowledge (*biomedical natural language processing* [*BioNLP*] corpus). Zhang et al [15] (*BioWordVec* corpus) and Flamholz et al [16] (*ClinicalEmbeddings* corpus) also leveraged PubMed and PubMed Central articles in addition to clinical notes from the MIMIC III to train embeddings using the FastText, GloVe, and Word2Vec algorithms [12].

### Word Embedding Evaluations

Systematic evaluations of word embeddings can be broadly classified into two categories, *intrinsic* and *extrinsic* evaluations. Intrinsic evaluations typically evaluate these word embeddings against human annotations by measuring the similarity or relationship between the queried and returned word pairs. Pakhomov et al [17,18] and Pedersen et al [19] have developed data sets containing pairs of biomedical terms along with their degree of relatedness as rated by human annotators. Furthermore, Pakhomov et al [17] and Hliaoutakis [20] have annotated pairs of medical terms for their semantic similarity. One intrinsic evaluation for validating these human annotations entails computing the Spearman coefficient between word pairs. Others have intrinsically evaluated word embeddings by clustering biomedical terms from the Unified Medical Language System and Ranker [21], and assessing the cluster quality using metrics like the Davies-Bouldin index and the Dunn index. Word embeddings have advanced the state of the art for many intrinsic natural language processing subtasks (ie, reading comprehension [22], natural language inference [23], text summarization [24], vocabulary development [8], and document classification [25]). An extrinsic or summative evaluation of clinical word embeddings can involve evaluating the performance of machine learning models by using word embeddings to complete a biomedical research task or clinical operation such as patient phenotyping [26,27], patient fall prediction [25], and patient hospital readmission prediction [28].

### COVID-19 and Word Embeddings

In recent years, there has been extensive work to leverage biomedical and clinical texts for developing clinical word embeddings to create concept lexicons [29]. For example, clinical word embeddings have been trained to identify drugs [30], substance abuse terms [8], and anatomical locations [16]. More recently, word embeddings have been used to understand the COVID-19 pandemic. For example, Schild et al [31] trained word2vec models for learning terms related to “virus” (“corona,” “covid,” “wuflu,” “coronovirus,” “coronavirus”) for understanding the emergence of sinophobic behavior on web communities like Twitter and 4chan’s /pol/ facing COVID-19 outbreaks. Klein et al [32] applied pretrained Bidirectional Encoder Representations from Transformers to identify Twitter users with probable or possible COVID-19 infection using their self-reported Twitter messages and temporal-spatial information. However, to our knowledge, there has been no intrinsic evaluation of openly available word embeddings to identify COVID-19 terms related to symptoms, findings, and disorder concepts for encoding clinical notes.

Our long-term goal is to develop a COVID-19 information extraction system to support a variety of purposes, including clinical and translational research, observational studies, clinical trials, public health monitoring, and hospital capacity monitoring. Our short-term goal is to conduct an *intrinsic evaluation* to qualitatively analyze and compare various openly available word embedding sources by categorizing the most similar words returned for symptoms, findings, and disorders related to COVID-19, and to identify common patterns between returned terms and their associated COVID-19 query terms to better understand which of these word embedding sources and their configurations could support synonym discovery. An additional short term goal is to conduct an *extrinsic evaluation* to apply these terms and their learned synonyms to the discharge summaries of patients with pneumonia, acute respiratory distress syndrome (ARDS), and COVID-19, and report the proportion of patients identified, with terms representing each concept for each disorder cohort.

## Methods

In this University of Pennsylvania Institute Review Board–approved study (#831895, #843620), we conducted a literature review of open-source word embeddings. We identified 7 publicly available sources and characterized each resource according to the training source, unit of processing, context window embedding technology, preprocessing, embedding technology used, returned units, embedding size, and vocabulary size (Table 1).

**Table 1:** Description of word embedding sources used.

Name	Author and source	Training source	Unit	Context window	Preprocess (reduce case, remove stop words, term types)	Embedding technology (gensim, FastText, GloVe^a^, BERT^b^, ELMO, etc)	Returned unit (1-3 ngrams)	Embedding size	Vocab size
BioNLP^c^ Lab PubMed + PMC^d^ W2V	Pyysalo et al 2013 [14,33]	PubMed/ PMC articles	Token	5	Mixed case, no stop words, skip-grams	word2Vec	1 ngram	200	~4 billion tokens
BioNLP LabWiki + PubMed + PMC W2V	Pyysalo et al 2013 [14,33]	Wikipedia, PubMed/ PMC articles	Token	5	Mixed case, no stop words, skip-grams	word2Vec	1 ngram	200	~5.4 billion tokens
BioASQ	Tsatsaronis et al 2015 [13,34]	PubMed abstracts	Token	5	Lowercase, no stop words, continuous bag of words	word2Vec	1 ngram	200	~1.7 billion tokens
Clinical Embeddings W2V300	Flamholz et al 2019 [16,35]	PubMed/ PMC/ MIMIC III^e^	Token	7	Lowercase, include stop words, skip-grams	word2Vec	1-3 ngrams	300	~300k tokens
BioWordVec Extrinsic	Zhang et al 2019 [15,36]	PubMed + MeSH^f^	Character	5	lowercase, include stop words	FastText	1-3 ngrams	200	~2.3 billion tokens
BioWordVec Intrinsic	Zhang et al 2019 [15,36]	PubMed + MeSH	Character	20	Lowercase, include stop words	FastText	1-3 ngrams	200	~2.3 million tokens
Standard GloVe Embeddings	Pennington et al 2014 [11]	Common Crawl	Token	10	Mixed case	GloVe	1 ngram	300	~2.1 billion tokens

^a^GloVe: global vectors for word representation.

^b^BERT: Bidirectional Encoder Representations from Transformers.

^c^BioNLP: biomedical natural language processing.

^d^PMC: PubMed Central.

^e^MIMIC III: Medical Information Mart for Intensive Care III.

^f^MeSH: Medical Subject Headings.

### Constructing the Reference Standard

We generated a list of terms for COVID-19–related semantic categories of *symptoms* (“fever,” “high fever,” “cough,” “wet cough,” “dry cough,” “congestion,” “nasal congestion,” “pain,” “chest pain,” “muscle pain,” “shortness of breath,” “dyspnea,” “tachypnea,” “malaise,” “headache,” “sore throat”), *findings* (“hypoxia,” “opacities,” “bilateral opacities,” “infiltrates,” “lung infiltrates”), and *disorders* (“ARDS,” “respiratory distress,” “acute respiratory distress syndrome,” “pneumonia”) described in Cascella et al [1]. We queried each word embedding source detailed in Table 1 using these COVID-19–related phrases and retrieved the top 20 phrases based on ranked cosine similarity (terms closest to 1.0 signifying high similarity). Three annotators (a biomedical informatician, a clinical general internist and informatician, and a second-year medical student) encoded each returned phrase with the following semantic class types:

- *Negation (black)*: a negation of the query term (eg, “afebrile” is a negation of “fever”)
- *Synonyms (green)*: a lexical variant of the query term with highly similar or synonymous meaning, including misspellings and short forms (eg, “ARDS” is a synonym for “Acute Respiratory Distress Syndrome”)
- *Symptom/signs (yellow)*: any symptom, observation, finding, or syndrome that is not a synonym of the query term (eg, “fever” is a symptom returned by “cough”)
- *Disease/disorders (blue)*: any disease, disorder, or diagnosis that is not a synonym for the query term (eg, “pneumonia” is a disorder returned by “dyspnea”)
- *Hyponym (light red)*: a more specific semantic type of the query term (eg, “ground-glass opacities” is a hyponym of “opacities”)
- *Hypernym (dark red)*: a broader semantic type of the query term (eg, “cough” is a hypernym of “productive cough”)
- *Qualifiers (teal)*: any nonclinical temporal, spatial, quality, extent, or size descriptor (eg, “dry” is a qualifier for “cough”)
- *Anatomical location (orange)*: any clinical anatomical or positional descriptor (eg, “lower lobe” is an anatomical location)
- *Therapeutic (purple)*: any medication, therapy, or procedure (eg, “mechanical ventilation” is a therapeutic device)
- *Other (grey)*: any semantic type that was not among the aforementioned or a nonclinical type (eg, “traffic” returned for “congestion”)

### Assessing Interannotator Agreement

For each annotator pair, we computed the interannotator agreement for the semantic class types for each queried term using Cohen kappa [37] using sklearn [38]. Specifically, for each queried phrase (eg, “fever”), each annotator encoded the *semantic type* of the returned candidate term compared to the queried term (eg, returned term “pyrexia” encoded as a *synonym* for queried term “fever”). We report the overall interannotator agreement by category (symptom, finding, and disorder) and by queried term (“fever,” “dry cough”). We also depict semantic disagreements between each pair of annotators using heat maps generated using matplotlib [39].

### Analyzing the Similarity Between COVID-19 Queried and Returned Terms

We depict the broad range of terms returned across openly available word embedding sources. For each queried term, the returned term will maintain the same semantic type across word embedding sources but might return a different cosine similarity or occur in only select sources. Therefore, for all unique returned terms within the top 20 ranked by cosine similarity, we visualized the returned term based on its frequency among the word embedding sources at any rank using word clouds generated with matplotlib. The size of the word is a weighted representation of how frequently the returned term occurred across the seven-word embedding source; the score is bounded between 0.14 (observed within only one of seven word embedding sources) and 1.0 (observed within all seven word embedding sources). Additionally, of the terms that occurred *at least once* among the top 20 ranked terms across the seven embeddings, we plotted the range of cosine similarities. Observed top-ranked terms may have cosine similarity values ranging from 0 to 1.0. If a top-ranked term was not found within another embedding source, the term received a value of –1.

### Assessing the Semantic Distribution Patterns for Returned Candidate Terms by Source

We determined the distribution of semantic classes among returned candidates for each queried term according to word embedding source. Our goal is to identify common semantic themes among the queried-returned term pairs that might be driven by the word embedding source construction. We performed a content analysis with simple mean comparisons for each semantic category as well as terms with and without modifiers across embedding sources to identify additional association patterns (Table 2).

**Table 2:** Queried terms (symptoms, findings, and disorders) with and without modifiers.

Category and term without modifier			With modifier
**Symptoms**			
	“fever”	“high fever”	
	“cough”	“wet cough,” “dry cough”	
	“congestion”	“nasal congestion”	
	“pain”	“chest pain,” “muscle pain”	
**Findings**			
	“opacities”	“bilateral opacities”	
	“infiltrates”	“lung infiltrates”	
**Disorders**			
	“ARDS”^a^	“respiratory distress,” “acute respiratory distress syndrome”	

^a^ARDS: acute respiratory distress syndrome.

### Generating Symptom Severity Profiles for Patients With Pneumonia, ARDS, and COVID-19

As a proof of concept, we compared the proportion of patients that can be classified according to COVID-19 illness severity groups using terms indicative of their clinical features for three cohorts: patients with *pneumonia*, *ARDS*, and *COVID-19*. For the patients with pneumonia and ARDS cohorts, we queried all inpatient encounters and their resulting discharge summaries with COVID-19–related disorders: ARDS (International Classification of Diseases [ICD] codes: 518.5, 518.81, 518.82) and pneumonia (ICD codes: 480-488) from the MIMIC III database [12]. For the patients with COVID-19 cohort, we queried all COVID-19 inpatient encounters from our EPIC PennChart COVID-19 registry from March 2020 to August 2020 and the resulting discharge summaries. In Table 3, we denote the clinical findings associated with COVID-19 respiratory illness severity categories [1]. We applied the expanded lexicon for COVID-19 respiratory illness severity clinical features using synonyms detected from all embedding approaches (*keywords + embedding expansion*). For each cohort, we report the proportion of patients with the clinical feature documented within one or more discharge summaries.

**Table 3:** Clinical findings according to the COVID-19 respiratory illness severity groups.

COVID-19 respiratory illness severity	Clinical features
Mild illness	Mild fever, cough (dry), sore throat, malaise, headache, muscle pain, nasal congestion
Moderate pneumonia	Cough and shortness of breath
Severe pneumonia/acute respiratory distress syndrome	Fever is associated with severe dyspnea, respiratory distress, tachypnea, and hypoxia

## Results

We queried seven embedding sources with 15 symptom terms, five finding terms, and four disorder terms, resulting in 10,080 annotations (top 20 returned candidate terms × 25 queried terms × seven word embedding sources × three annotators).

### Assessing Interannotator Agreement

**Figure 1:**
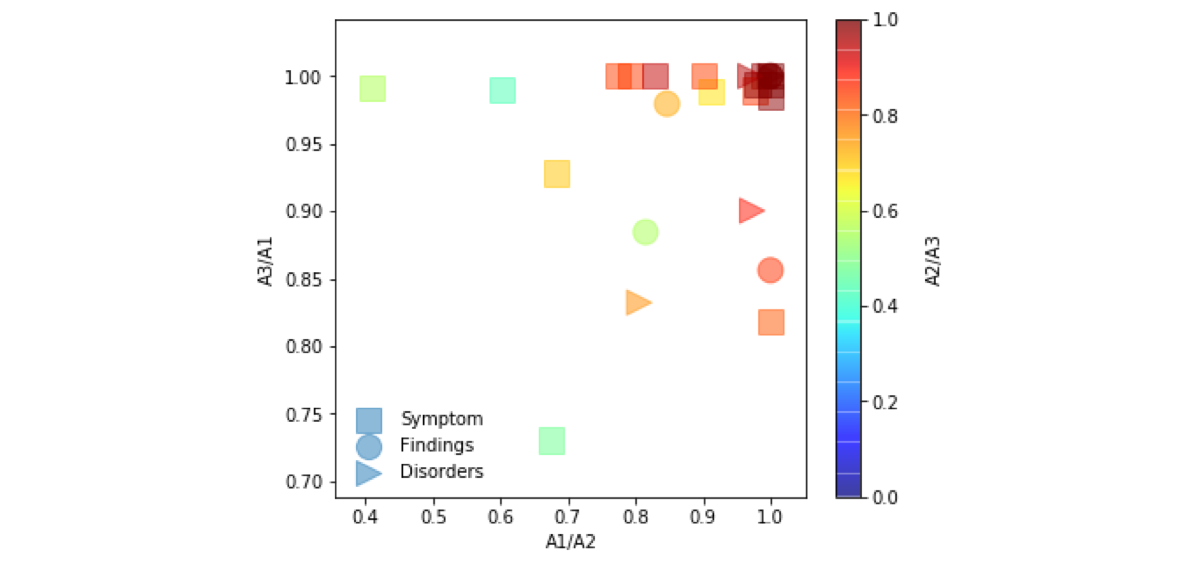
Pairwise interannotator agreement according to semantic category for each queried term.

We observed high overall pairwise interannotator agreement between annotators (ie, A#=Annotator#) for each semantic category: symptoms (0.86-0.99), findings (0.93-0.99), and disorders (0.93-0.99). For A1/A2 and A2/A3, we observed low to moderate interannotator agreement for “malaise” (0.40-0.41), “muscle pain” (0.6), “headache” (0.65-0.68), and “dry cough” (0.68). For A3/A1, interannotator agreement was consistently high (≥0.93). In Figure 1, we report the distribution of each queried term’s overall agreement between paired annotators. The color bar represents the third annotator pair. Overall agreement by COVID-19 category and by queried term for each annotator pair can be found in Multimedia Appendix 1.

**Figure 2:**
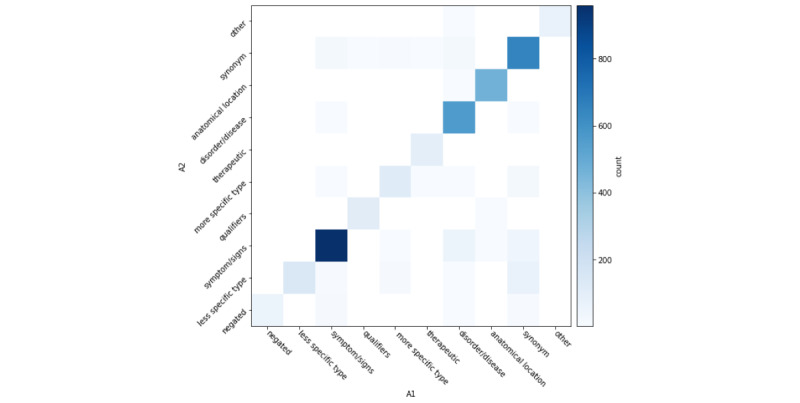
A1/A2 interannotator agreement of returned terms according to semantic type.

**Figure 3:**
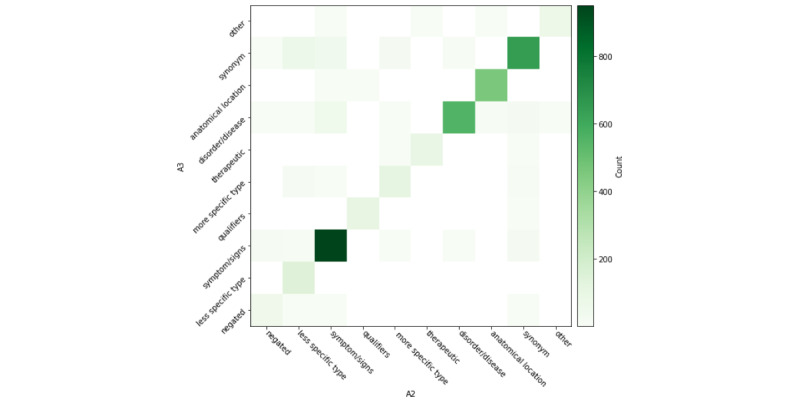
A2/A3 interannotator agreement of returned terms according to semantic type.

**Figure 4:**
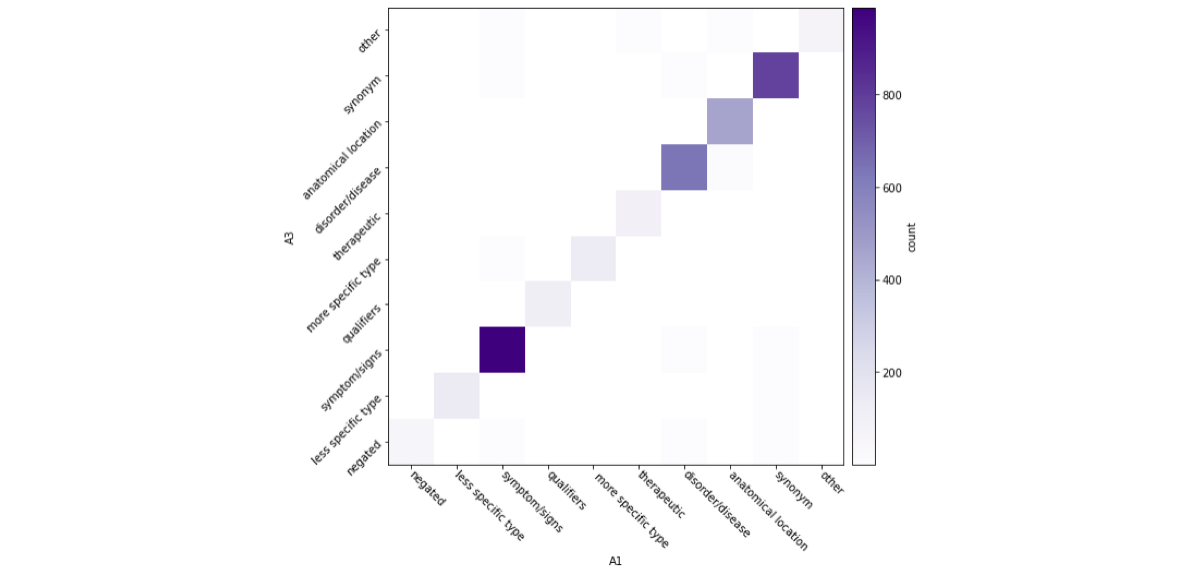
A1/A3 interannotator agreement of returned terms by semantic type.

In Figures 2-4, for each returned term, we also computed interannotator agreement across semantic types. Across annotator pairs, we observed high interannotator agreement for all semantic types. Each heat map depicts systematic differences between annotators. In Figure 2, A1/A2 more often disagreed about whether a returned term was a hypernym, hyponym, or negation. In Figure 3, A2/A3 more often disagreed about whether a returned term was a synonym, disease or disorder, hypernym, hyponym, other, or negation. In Figure 4, A3/A1 most often disagreed about whether a returned term was a negation or other term.

### Analyzing the Similarity for Returned Candidate Terms

**Figure 5:**
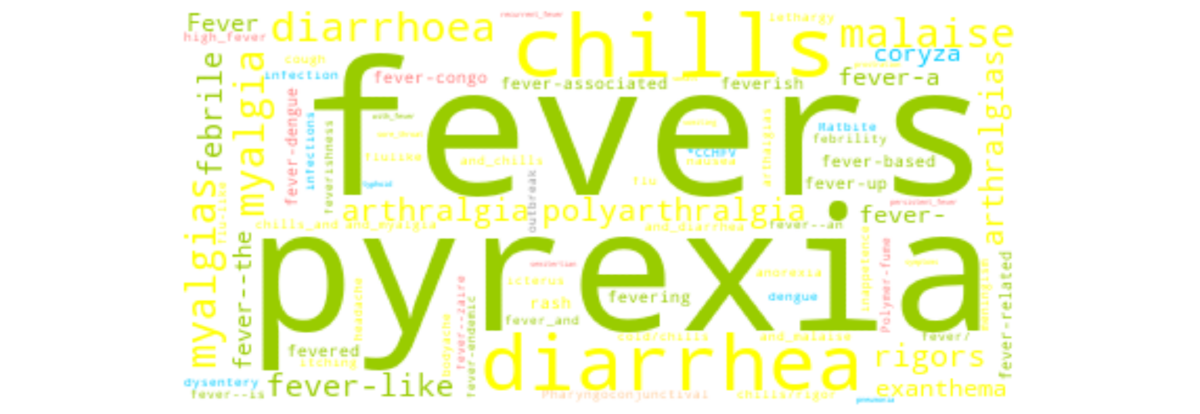
Word cloud depicting each returned term for “fever.” Colors correspond to semantic class types.

**Figure 6:**
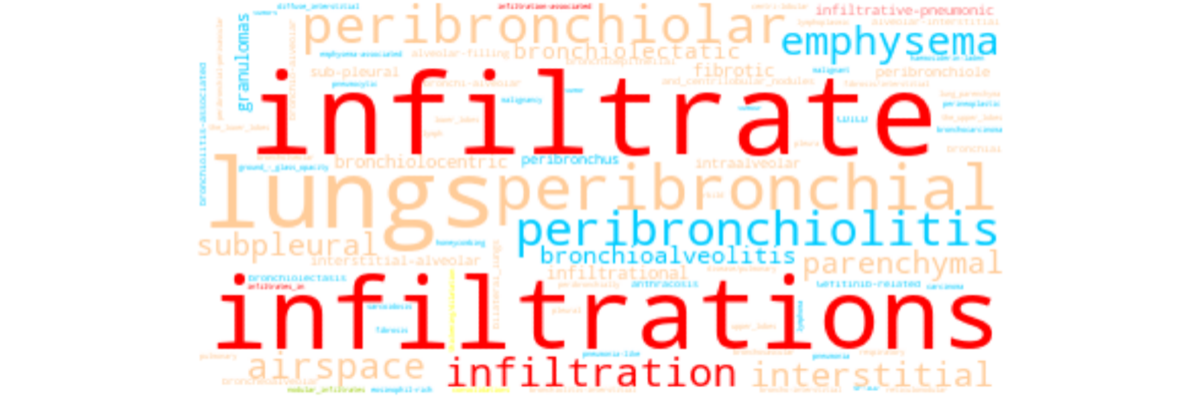
Word cloud depicting each returned term for “lung infiltrates.” Colors correspond to semantic class types.

**Figure 7:**
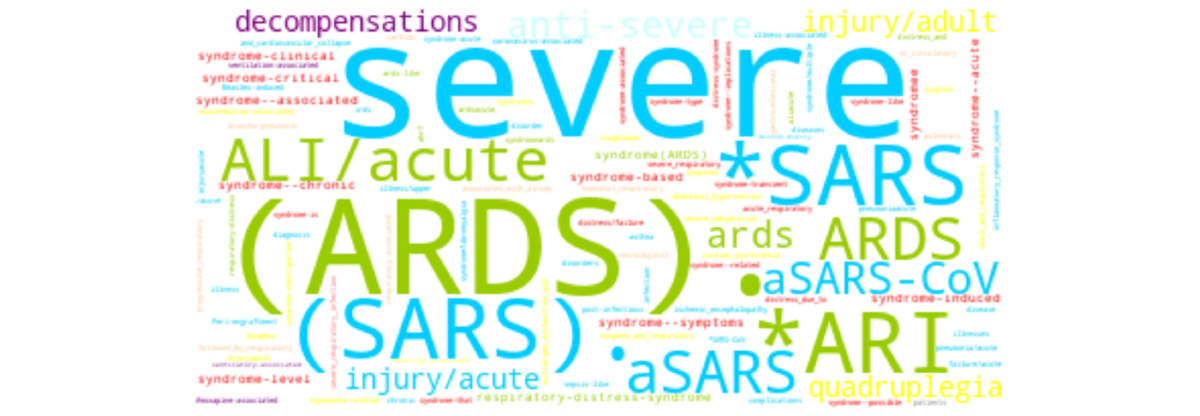
Word cloud depicting each returned term for “acute respiratory distress syndrome.” Colors correspond to semantic class types. ALI: acute lung injury; ARDS: acute respiratory distress syndrome; ARI: acute respiratory infection; SARS: severe acute respiratory syndrome; SARS-CoV: severe acute respiratory syndrome–related coronavirus.

We report the broad range of queried terms returned across word embedding sources. For brevity, we depict three COVID-19–related concepts, one of each semantic category: *symptom* (“fever”; Figure 5), *finding* (“lung infiltrates”; Figure 6), and *disorder* (“acute respiratory distress syndrome”; Figure 7). For “fever,” synonyms (eg, “pyrexia,” “fevers,” and “febrile”) and signs or symptoms (eg, “chills” and “diarrhea”) were common among the returned terms. For “lung infiltrates,” the most frequent semantic types included anatomical locations (eg, “lungs,” and “peribronchial”) and hypernyms (eg, “infiltrate” and “infiltration”) were among the returned terms. For “ARDS,” disease or disorders (eg, “SARS” [severe acute respiratory syndrome] and “aSARS-CoV”), synonyms (eg, “ards” and “respiratory-distress-syndrome”), and hypernyms (eg, “syndromee” and “syndrome-critical” were observed commonly among the returned terms.

**Figure 8:**
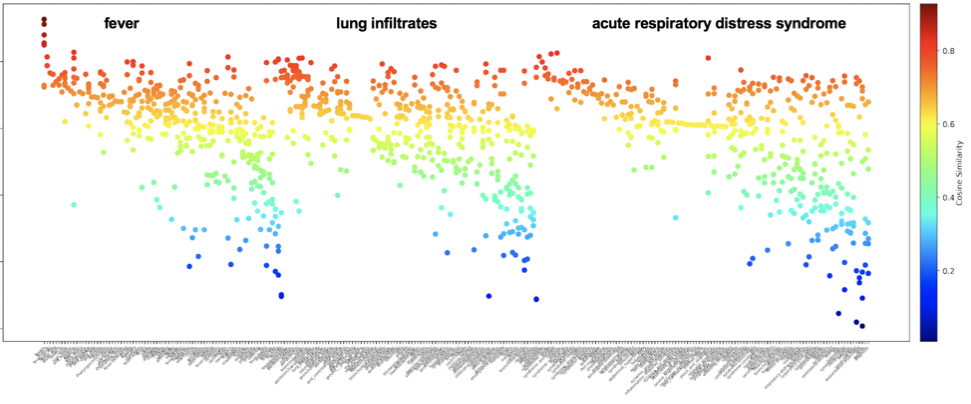
Cosine similarity measures for each unique returned term among the top 20 terms across all word embedding sources returned for the queried terms “fever,” “lung infiltrates,” and “acute respiratory distress syndrome.” Color range indicative of cosine similarity level (0.0-1.0), not semantic type.

In Figure 8, we observe that, given a queried term (eg, “fever,” “lung infiltrates,” and “acute respiratory distress syndrome”), returned terms differ by cosine similarity and variance. For example, some returned terms have high cosine similarity and low variability (left most in red and orange only), while others demonstrate variable cosine similarity and high variability (right most in all colors). Examples of returned terms with *high cosine similarity and low variability* include “fever”: “fevers,” “fevering,” and “pyrexia”; “lung infiltrates”: “infiltration,” “infiltrates,” and “peribronchial”; and “acute respiratory distress syndrome”: “syndrome(ARDS),” “aSARS,” and “syndromeards.” Examples of returned terms with *variable cosine similarity and high variability* include “fever”: “fevered,” “fever-based,” and “fever-like”; “lung infiltrates”: “infiltrational,” “consolidations,” and “bronchioepithelial”; and “acute respiratory distress syndrome”: “syndrome-is” and “syndrome-level.”

### Assessing the Distribution of Semantic Types for Returned Candidate Terms by Source

**Figure 9:**
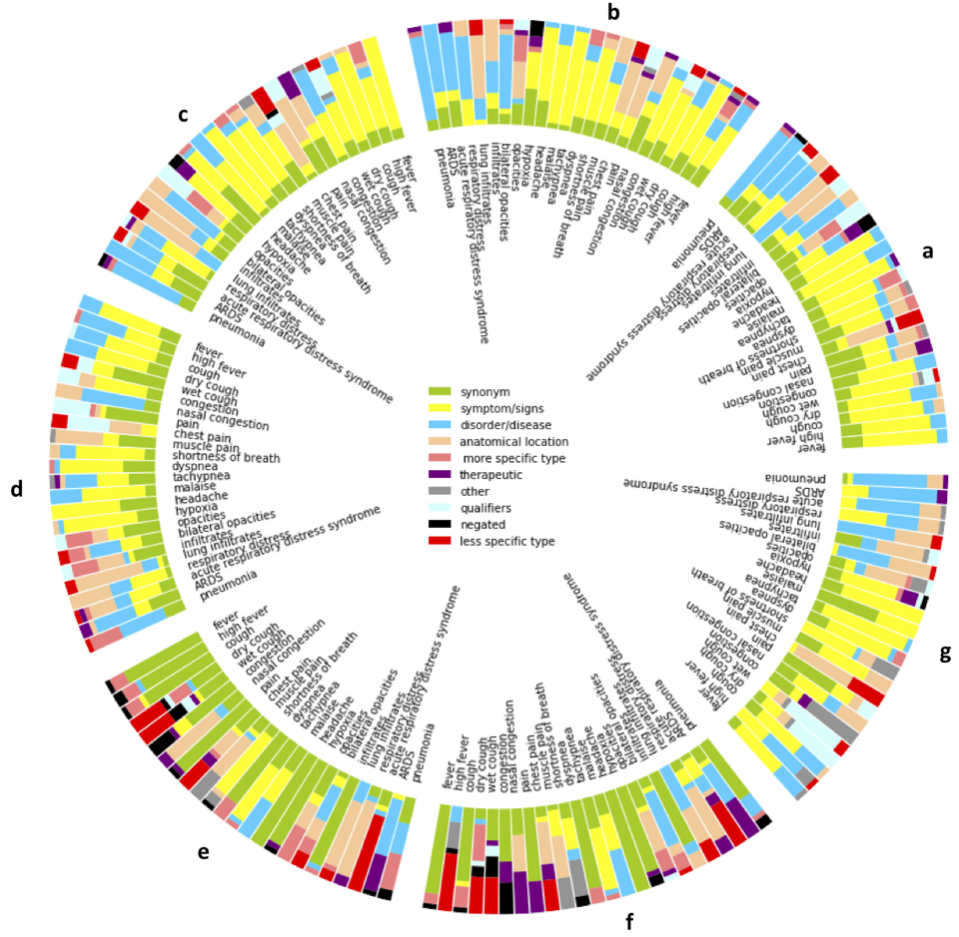
For each symptom, finding, and disorder queried term, the distribution of semantic types for returned term colored by semantic type for each embedding source: (a) BioNLP Lab PubMed + PMC W2V, (b) BioNLP LabWiki + PubMed + PMC W2V, (c) BioASQ, (d) Clinical Embeddings W2V300, (e) BioWordVec Extrinsic, (f) BioWordVec Intrinsic, and (g) Standard GloVe Embeddings. ARDS: acute respiratory distress syndrome.

We determined the distribution of semantic classes among returned candidates for each queried term according to word embedding source. Our goal is to identify common semantic themes among the queried and returned candidate term pairs that might be driven by the word embedding source construction. We observed that the *BioWordVec Extrinsic* and *BioWordVec Intrinsic* embeddings (Figure 9 e-f) were more likely to generate synonyms (green), which is notably depicted for “fever,” “headache,” “hypoxia,” “dyspnea,” and “infiltrates.” Word embedding sources generated based on characters tend to return more synonyms (mean count of 7.2 synonyms) compared to token-based embedding sources (mean count ranged from 2.04 to 3.4 synonyms). We also observed more negation terms for “hypoxia” (mean count of 2.29 negations); “congestion” (mean count of 1.57 negations); and “dry cough,” “wet cough,” and “tachypnea” (all had mean counts of 1.0 negations) compared to other terms (mean counts ranged from 0.00 to 0.71 negations). We observed a high mean count of hypernyms for “dry cough” (mean count of 6.43 hypernyms), “high fever” (mean count of 5.57 hypernyms), and “acute respiratory distress syndrome” (mean count of 4.43 hypernyms) over other terms (mean counts ranged from 0 to 3.29 hypernyms). Across the other word embeddings (Figure 9 a-d and g), if a symptom or sign queried term was provided, we more often observed a symptom or sign returned term (mean average of 6.62 symptoms or signs) compared to nonsymptom or sign queried terms (mean average of 3.035 symptoms or signs). This also held true for disorders (mean average of 6.24 disorders) compared to nondisorders (mean average of 1.18 disorders). Across word embedding sources (Figure 9), we observed that qualifiers were more often returned when the queried term contained a qualifier for some terms (eg, “dry cough” and “wet cough” return time and consistency qualifiers like “wet” and “runny”; both mean counts of 4.14 qualified terms) over the nonqualified queried term “cough” (mean count of 1.71 qualified terms). Similar patterns were observed for “high fever” (mean count of 3.71 qualified terms), “fever” (mean count of 0.0 qualified terms), “bilateral opacities” (mean count of 6.14 qualified terms), and “opacities” (mean count of 2.71 qualified terms). Furthermore, if a queried term contained an anatomical location as an adjective in the term phrase (eg, “nasal congestion”), the returned terms were often anatomical locations compared to queried terms without adjectives. We observed notable differences in mean counts of returned terms with anatomical qualifiers for “nasal congestion” (mean count of 6.71 anatomical terms) and “congestion” (mean count of 0.42 anatomical terms), “chest pain” (mean count of 8.43 anatomical terms) and “pain” (mean count of 3.57 anatomical terms), and “lung infiltrates” (mean count of 10.57 anatomical terms) and “infiltrates” (mean count of 6.71 anatomical terms). In few cases, the *standard GloVe embeddings*, *BioWordVec Extrinsic*, and *BioWordVec Intrinsic* embeddings returned some terms with common term usage (eg, “congestion” returns “traffic,” “bypass,” or stop words such as “and,” “a,” and “of”).

### Generating Symptom Severity Profiles for Patients With Pneumonia, ARDS, and COVID-19

**Figure 10:**
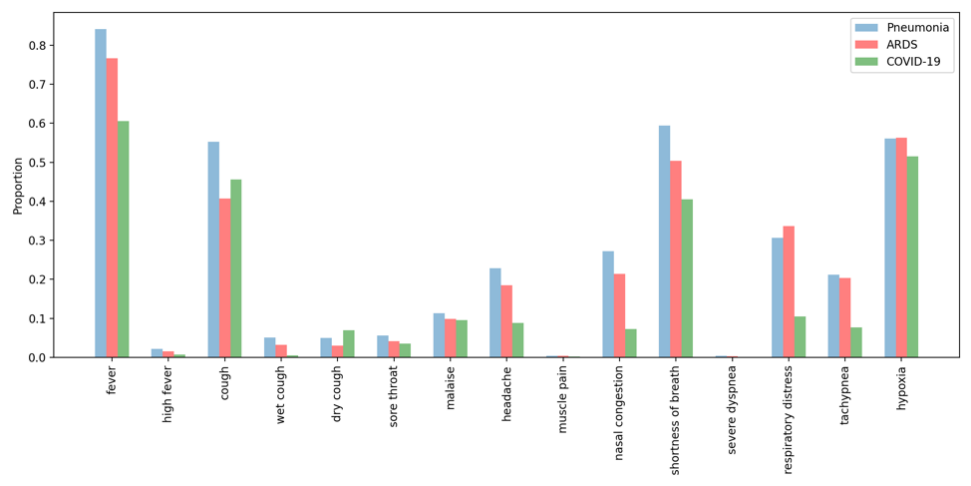
The proportion of patients with each COVID-19 clinical feature documented within their discharge summary according to disorders (pneumonia, ARDS, and COVID-19). ARDS: acute respiratory distress syndrome.

Figure 10 shows the proportion of patients from each disorder cohort (pneumonia, ARDS, and COVID-19) that have one or more terms documented within their discharge summary representing clinical features from Table 3. The total number of patients in each cohort varied: pneumonia (n=6410), ARDS (n=8647), and COVID-19 (n=2397). A higher proportion of patients had documented fever (0.61-0.84), cough (0.41-0.55), shortness of breath (0.40-0.59), and hypoxia (0.51-0.56) retrieved than other clinical features. Terms for dry cough returned a higher proportion of patients with COVID-19 (0.07) than pneumonia (0.05) and ARDS (0.03) populations.

## Discussion

### Assessing Interannotator Agreement

We observed high overall pairwise interannotator agreement for the symptoms, findings, and disorder categories. Annotators A1 and A3 were more often in agreement. For the A1/A2 and A2/A3 pairs, we observed low to moderate interannotator agreement for queried terms such as “malaise,” “muscle pain,” “headache,” and “dry cough.” Annotators A1 and A3 systematically classified notably fewer returned terms as hypernyms and hyponyms than A2. For example, “migraine” is a hypernym for “headache.” Additionally, A2 more easily identified negated terms through medical terminology. Many cases required more clinical domain knowledge to make these distinctions, which were easier for the general internist.

### Analyzing the Similarity Between COVID-19 Queried and Returned Terms

When analyzing the cosine similarities between queried terms and returned terms, we observed that returned terms range from *high cosine similarity and low variability* to *variable cosine similarity and high variability*. We hypothesize that terms with high cosine similarity and low variability are more likely to be synonyms and useful for training an information extraction. In practice, the presence and cosine similarities of a term varied across word embedding sources. Our ability to identify and rank likely synonyms for lexicon development may be improved with additional processing steps and comparisons between the queried and returned terms for lexical similarity [40], morphological derivation [8], and short form construction and expansion [41].

### Assessing the Semantic Distribution Patterns for Returned Candidate Terms by Source

We determined the distribution of semantic classes among returned candidates for each queried term according to the word embedding source. Our study intentions were to assess the distributional hypothesis that words with similar meanings are often used in similar contexts. Generally, if a symptom or sign queried term was provided, we often observed a symptom or sign returned term. This also held true for disorders. Furthermore, our goal was to identify common semantic themes among the queried and returned candidate term pairs that might be driven by the word embedding source construction. We observed that the *BioWordVec Extrinsic* and *BioWordVec Intrinsic* embeddings were more likely to generate synonyms. We hypothesize that this is likely due to training based on the characters rather than the token; thus, the returned terms often share a common set of characters (queried term: “*fever*”; returned term: “*fever*ish”) or high lexical similarity. Character-based embeddings will often return lexical variations of the queried term. Although *BioNLP*, *BioASQ*, and *Clinical Embeddings* generated fewer synonyms, these were often medical terms for the lay queried term (eg, “lethargy” for “malaise,” “cephalea” for “headache,” and “rhinorrhea” for “nasal congestion”). To maximize the diversity of learned synonyms, multiple embeddings could be most beneficial. Returned negated terms were expressed with prefixes (eg, “non-pneumonia-related”), suffixes (eg, “fever-free”), or medical terminology (eg, “normoxia”). Hypernyms were commonly observed among queried terms with an adjectival phrase (eg, “high fever,” “muscle pain,” “dry cough,” and “lung infiltrates”). Moreover, we observed that qualifiers were often returned when the queried term contained a qualifier (eg, time, consistency, and anatomical location qualifiers). For developing a clinical information extraction system, these returned terms can be useful for brainstorming synonyms as inclusionary terms as well as antonyms as exclusionary terms. We suspect that a mix of hypernyms and qualifiers were often returned, given the semantics of the individual parts of the queried phrase. It was not surprising that *standard GloVe* embeddings returned some terms with a nonclinical word sense (eg, “congestion” returns “traffic” or “bypass”) because they were trained using the CommonCrawl domain-independent corpora. Similarly, *BioWordVec Extrinsic* and *BioWordVec Intrinsic* occasionally return stop words, as these were not removed prior to training and perhaps should be for detecting meaningful synonyms.

### Generating Symptom Severity Profiles for Patients With Pneumonia, ARDS, and COVID-19

We created an expanded lexicon of COVID-19 respiratory illness clinical features (Table 3) using synonyms detected from all embedding approaches. We assessed the proportion of patients from three disorder cohorts (pneumonia, ARDS, and COVID-19) with each clinical feature documented within their discharge summary. We observed that terms indicative of clinical features for fever, cough, shortness of breath, and hypoxia retrieved a higher proportion of patients than clinical features. For fever and cough, our lexicons for capturing contextualized mentions of these clinical features (eg, high fever or wet or dry cough) retrieved modest proportions of patient cases. This is likely due to the variability of qualitative and quantifications of these symptoms (eg, productive cough and fever of 102° F) in discharge summaries. Terms indicative of dry cough returned a higher proportion of patients with COVID-19 than pneumonia and ARDS populations. This is not surprising given that this is a prominent symptom reported among patients with COVID-19.

### Limitations and Future Work

Our study has a few notable limitations. We began this study during the early stages of the COVID-19 pandemic when the symptomatology was less understood. COVID-19 is a heterogeneous disease with emerging symptomatology identified through ongoing clinical observational studies. Emerging COVID-19–related symptomatology (ie, loss of smell, loss of taste, and COVID toes) were not included in our analysis, as their association with COVID-19 were not well understood at the time of our study. We leveraged existing word embedding sources to better understand the utility of embeddings for synonym generation. We recognize that further experimentation is needed to support broader claims of their utility. As a proof of concept of patient information retrieval, we applied an expanded lexicon of terms representing clinical features of COVID-19 to three disorder cohorts (pneumonia, ARDS, and COVID-19). Although these terms retrieved a high proportion of patients, we acknowledge that additional terms might be necessary to accurately identify these features and that contextualization (ie, negation, severity, experiencer, and temporality [42-44]) is critical to generating accurate patient profiles. We look forward to addressing these issues as next steps within our clinical information extraction pipeline powered by Linguamatics [45]. These text-derived and contextualized variables will be available through our clinical research databases—COVID-19 Informatics for Integrating Biology and the Bedside database [46] and Penn Genotype and Phenotype database supported by the Observational Medical Outcomes Partnership common data model [47]—at the end of Spring 2021.

### Conclusion

Word embeddings are a valuable technology for learning semantically and syntactically related terms including synonyms and useful for text classification and information extraction tasks. When leveraging openly available word embedding sources, choices made in the development of the embeddings can significantly influence the types of phrases and information learned.

## Appendix

Multimedia Appendix 1 Overall agreement by COVID-19 category and by queried term for each annotator pair.

## Abbreviations

ARDS: acute respiratory distress syndrome
BioNLP: biomedical natural language processing
GloVe: global vectors for word representation
ICD: International Classification of Diseases
MIMIC III: Medical Information Mart for Intensive Care III
SARS: severe acute respiratory syndrome
