# Supplementary material for: Lexicon Development for COVID-19-related Concepts Using Open-source Word Embedding Sources: An Intrinsic and Extrinsic Evaluation
Source: JMIR Med Inform. 2021 Feb 22;9(2):e21679. doi: 10.2196/21679 (PMC7901592; doi:10.2196/21679)
Supplement: Multimedia Appendix 1 [file medinform_v9i2e21679_app1.docx]

**Table 1.** Overall agreement by COVID19 category and by queried term for each annotator pair

| **Category** | **Queried Term** | **Annotator1/**  **Annotator2** | **Annotator2/**  **Annotator3** | **Annotator3/Annotator1** |
| --- | --- | --- | --- | --- |
| **Symptoms** |  | 0.87 | 0.86 | 0.99 |
|  | fever | 0.91 | 0.89 | 0.98 |
|  | ***high fever*** | ***0.77*** | ***0.77*** | ***1.0*** |
|  | cough | 0.90 | 0.90 | 1.0 |
|  | ***dry cough*** | ***0.68*** | ***0.68*** | ***0.93*** |
|  | wet cough | 1.0 | 1.0 | 1.0 |
|  | congestion | 1.0 | 0.98 | 0.98 |
|  | ***nasal congestion*** | ***1.0*** | ***0.97*** | ***0.97*** |
|  | pain | 0.83 | 0.83 | 1.0 |
|  | ***chest pain*** | ***0.98*** | ***0.99*** | ***0.99*** |
|  | ***muscle pain*** | ***0.60*** | ***0.60*** | ***0.98*** |
|  | shortness of breath | 0.98 | 0.95 | 0.98 |
|  | ***dyspnea*** | ***1.0*** | ***0.99*** | ***0.99*** |
|  | tachypnea | 0.79 | 0.79 | 1.0 |
|  | ***malaise*** | ***0.41*** | ***0.40*** | ***0.99*** |
|  | *headache* | *0.68* | *0.65* | *0.97* |
|  | ***sore throat*** | ***0.99*** | ***0.99*** | ***1.0*** |
| **Findings** |  | 0.94 | 0.93 | 0.99 |
|  | ***hypoxia*** | ***0.81*** | ***0.80*** | ***0.99*** |
|  | opacities | 1.0 | 1.0 | 1.0 |
|  | ***bilateral opacities*** | ***0.85*** | ***0.80*** | ***0.96*** |
|  | infiltrates | 1.0 | 1.0 | 1.0 |
|  | ***lung infiltrates*** | ***1.0*** | ***0.99*** | ***0.99*** |
| **Disorders** |  | 0.94 | 0.93 | 0.99 |
|  | respiratory distress | 0.97 | 0.97 | 1.0 |
|  | ***acute respiratory distress syndrome*** | ***0.97*** | ***0.94*** | ***0.97*** |
|  | ARDS | 0.80 | 0.79 | 0.99 |
|  | ***pneumonia*** | ***1.0*** | ***1.0*** | ***1.0*** |
